# Data Downlink System in the Vast IOT Node Condition Assisted by UAV, Large Intelligent Surface, and Power and Data Beacon

**DOI:** 10.3390/s20205748

**Published:** 2020-10-10

**Authors:** Zhibo Zhang, Qing Chang, Na Zhao, Chen Li, Tianrun Li

**Affiliations:** School of Electronic and Information Engineering, Beihang University, Beijing 100191, China; zhangzhibo94@buaa.edu.cn (Z.Z.); na_zhao@buaa.edu.cn (N.Z.); tonylc@buaa.edu.cn (C.L.); ltr@buaa.edu.cn (T.L.)

**Keywords:** IOT, UAV, LIS, PDB, trajectory planning, minimum energy consumption, minimum time consumption

## Abstract

The future development of communication systems will create a great demand for the internet of things (IOT), where the overall control of all IOT nodes will become an important problem. Considering the essential issues of miniaturization and energy conservation, in this study, a new data downlink system is designed in which all IOT nodes harvest energy first and then receive data. To avoid the unsolvable problem of pre-locating all positions of vast IOT nodes, a device called the power and data beacon (PDB) is proposed. This acts as a relay station for energy and data. In addition, we model future scenes in which a communication system is assisted by unmanned aerial vehicles (UAVs), large intelligent surfaces (LISs), and PDBs. In this paper, we propose and solve the problem of determining the optimal flight trajectory to reach the minimum energy consumption or minimum time consumption. Four future feasible scenes are analyzed and then the optimization problems are solved based on numerical algorithms. Simulation results show that there are significant performance improvements in energy/time with the deployment of LISs and reasonable UAV trajectory planning.

## 1. Introduction

Urban deployment of the Internet of Things (IOT) with the characteristics of high efficiency and high security will be realized in the near future [1,2]. All devices will become IOT nodes and will be controlled by the cloud, and because of their intelligence, smart cities will be formed [3]. Vast IOT nodes are used to gather and disseminate information, and all the nodes need to collect information from the communication system to realize the overall collaboration. In order to save costs for the entire system, miniaturization and energy conservation of the nodes have become pressing issues.

Radio frequency energy transfer (RFET) is a new widely researched technology [4], where energy in the form of electromagnetic radiation is carried by radio signals and collected by receivers. Charging a system wirelessly can achieve a full-time operation and avoid the cost of wired charging circuits. Then, if the devices of all IOT nodes get rid of such circuits, a lot of space can be saved. This is beneficial as it allows for the miniaturization of the IOT system. Depending on whether a system relies on energy-receiving devices storing energy or directly reflecting it, RFET systems can be categorized into twotypes: energy-harvesting systems and backscattering systems [5,6]. Energy harvesting systems can be used for both downlink and uplink communication, while backscattering systems are often used for uplink communication [7].Thus, by using RFET technology and building an energy-harvesting system, IOT nodes can acquire enough energy to complete the downlink data receiving procedure.

When being integrated into communication systems, unmanned aerial vehicles (UAVs) are often regarded as aerial base stations (ABSs), relay platforms, and/or user equipment [8]. As aerial communication platforms, UAVs play roles as assistants to terrestrial communication systems and/or just as cellular-connected devices. Firstly, ABSs provide the system with advantages, such as swift deployment and high line-of-sight (LOS) communication probability. They are of great help when building a communication network in temporary situations or specific scenes, such as during rapid network recovery and in dense communication user networks [9,10]. Further, with the ability to fly directly over the users, the LOS communication probability increases and, therefore, communication stability is improved [11]. In addition, it is convenient and flexible that ABSs can be deployed almost anywhere in a city, and UAVs can be regarded as sources of downlinks. Thus, in the considered scene of serving terrestrial IOT nodes, UAVs can replace ground base stations (GBSs) and save space on the ground, and then the entire system is miniaturized. When a UAV serves in the scene of vast IOT nodes, all information needs to be transferred to all of the IOT nodes from antennas on the UAV. In order to increase the channel capacity (CP), several transmitting antennas often exist [12].

Using large intelligent surfaces (LISs) is another way to enlarge the CP. These have been developed in recent years and will undergo mass deployment in the near future [13]. Most previous research has focused on the channels among GBSs, LISs, and users [14,15,16]. By applying reflector arrays, which are cheap and passive artificial structures on the outside walls of buildings, transmission signals are reflected, and then auxiliary communication channels are built. An auxiliary channel contains three major components: an information source unit, an information home unit, and a reflector-array-type LIS. The LIS consists of many phase shifters, and the phase-shifting values are determined by the incidence and reflection angles. By setting the values reasonably, a maximal CP can be reached [15]. Because there are no essential differences between GBSs and ABSs, it is feasible to apply LISs to facilitate communication from UAVs to ground devices (i.e., IOT nodes or PDBs). Then, auxiliary channels are also generated and contribute to downlink data transmission.

The above works illustrate the feasibility of technologies, such as FRFT, UAV communication, and LIS. All of these technologies dramatically enhance the performance of a communication system by continuous energy provision, flexible system deployment, or stable CP enhancement. There will be a common scenario in future communication systems where UAVs, IOTs, and LISs are widely deployed in smart cities. However, to the best of our knowledge, few studies have considered the integrated utilization of these technologies. In [17], a system model in which IOT nodes are assisted by a UAV and an LIS to communicate with a base station is proposed. Nevertheless, the charging procedure of IOT nodes was not considered, limiting the system’s endurance. In [18], a UAV-enabled wireless power transfer system was designed to address the trajectory planning problem and maximize the harvested energy of IOT nodes, but the data transmission procedure was not included. In [7], a UAV using backscattering technology was described. This acts as a data collector in an uplink communication system, but is unsuitable for use in a data downlink system. Thus, it is necessary to research an integrated UAV downlink communication system that includes both the wireless energy transfer procedure and the data transmission procedure and to determine how the whole system can achieve optimal system performance.

In the UAV downlink communication system considered in this paper, a UAV provides energy and downlink data to all IOT nodes, which are settled between two buildings. In addition, LISs are configured on both of the two buildings. If the UAV charges all IOT nodes directly, the system is infeasible for the following two reasons: Firstly, if an omnidirectional antenna is used to spread the energy waves, due to the existence of path loss, the receiving power will be quite low and the charging time will be quite long. Secondly, if a directional antenna is used to increase the charging transmission gain, the most important problem is that the UAV must know the exact positions of all IOT nodes, and it is also unreachable in the vast IOT node condition.

To tackle the above problem, power and data beacons (PDBs) can be deployed as relay stations for charging and data transmission. A widely used model of all of the IOT nodes is the Matern cluster process, in which all nodes are randomly distributed around all PDBs with a certain expectation for the node number [19]. The PDBs play a role in determining who receives the energy and downlink data and then transmits energy and data to all IOT nodes nearby. In addition, because the positions of PDBs are known, a directional antenna can be chosen to allow the UAV to deliver the energy.

**Figure 1:**
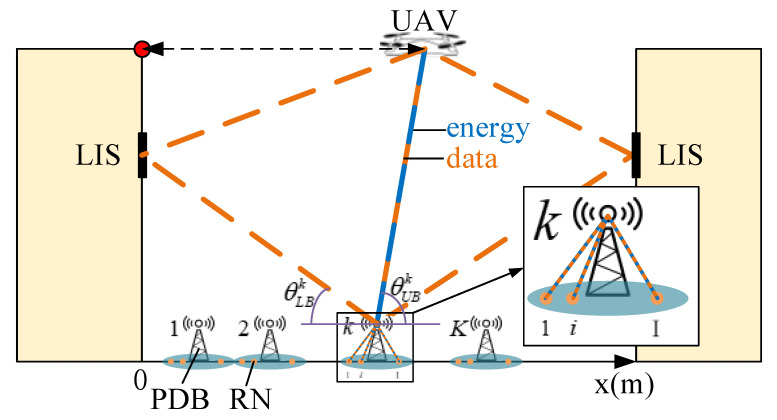
The scene with unmanned aerial vehicles (UAVs), the internet of things (IOT), and large intelligent surfaces (LISs).

The scene mentioned above is shown in Figure 1. The downlink process was researched and all IOT nodes receive energy and data. All of the IOT nodes are called receiving nodes (RNs), below. The entire system operates via time-division multiple access (TDMA), so the UAV connects all PDBs ordeDB connects nrly and each Pearby RNs orderly. At each time slot, there are three data transmitting paths or one energy delivering path between the UAV and a PDB, and there is one data transmitting path or one energy delivering path between a PDB and an RN. The UAV starts from the top of the left building and flies between the two buildings to complete the downlink data transmitting task. Obviously, the most valuable problem is to determine the optimal flight trajectory of the UAV to attain minimal energy consumption or minimal time consumption. This also conforms to the miniaturization and energy conservation of the entire communication system.

The main contributions of this paper are summarized below:

- A vast IOT node condition is considered in this paper, and to adapt to the above environment, the PDB device is innovatively proposed for energy and data relay.
- An integrated communication system model that contains both the data transmission procedure and the energy transfer procedure is designed in which the energy and data transfer process is innovatively designed as UAV–(LISs)–PDBs–RNs, and the entire system operates via TDMA.
- The task of the data downlink system is to transfer a certain number of data bits to all RNs, and the analytic expressions of the total energy cost and total time cost are derived. Two optimization problems are proposed to minimize the energy cost or the time cost. Trajectory planning is achieved by solving the proposed optimization problems, and all of them are solved by numerical algorithms, as with the linear search method and genetic algorithm.
- Four typical actual scenes are discussed, and their trajectory planning problems are solved. Simulation results are presented to examine the algorithms’ effectiveness and to show the significance of reasonable trajectory planning and the deployment of LISs.

The paper is organized as follows. In Section 2, the basic technology related to the PDB design, LIS, and wireless channel models is described. In Section 3, the data transmission and energy delivery models are presented, and optimization problems related to minimizing the total energy cost and time cost are proposed. In Section 4, the proposed optimization problems are solved by numerical algorithms, and the simulation results prove the performance improvement that results from deploying LISs and planning the UAV trajectory. In Section 5, the paper is concluded and a discussion of future research is given.

*Notation:* The variables, vectors, and matrices are denoted in italics *z*, bold italics z, and bold capital italics Z, respectively. The notation $∥•∥$ represents the Frobenius norm operator. The notation $E\left\{•\right\}$ represents the expectation of a random variable. The notation ${\left[•\right]}^{H}$ denotes the Hermitian transpose operator. The notation $\bar{•}$ indicates that a sub-channel is LOS, and $\tilde{•}$ represents NLOS. The notation $\overset{\Delta }{\;=}$ represents a definition of a formula. In addition, we use specific notations for different quantities—for example, *C* denotes the CP, *P* denotes the transmitting power or receiving power, *p* denotes the constant power or the probability, η or ξ denote the energy remaining ratios, *T* denotes the time cost, and *E* denotes the energy cost.

## 2. Basic Technology

### 2.1. Framework of PDB

**Figure 2:**
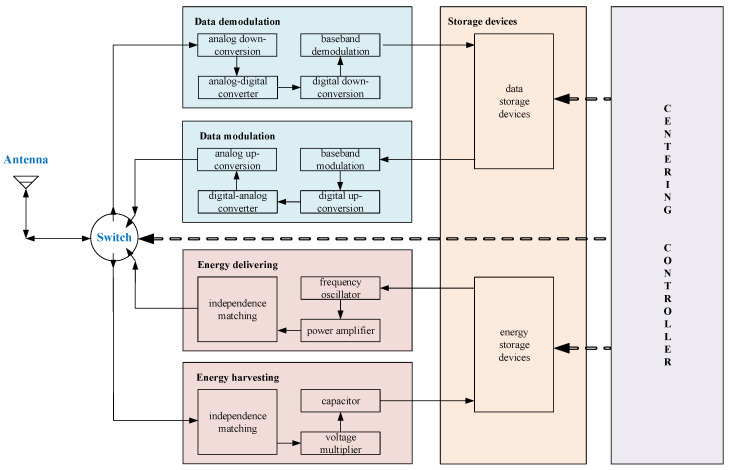
The structure of a power and data beacon (PDB).

All PDBs have four major functions: energy harvesting, data receiving, energy delivery, and data transmission. Thus, PDB devices must contain two parts of circuits: one for energy and the other for data. A typical framework of a PDB is illustrated in Figure 2 [5,20,21].

The PDB discussed in this paper only includes an antenna, and the four functions are chosen by a switch controlled by the centering controller. The single-channel design provides PDBs with two advantages: simple construction and low cost. In addition, due to the fact that the four functions form a fixed sequential circuit, no conflicts of the functions will occur when PDBs operate in a TDMA system. Then, when a PDB detects the energy transmitting signal from the UAV, the circulation of functions will operate automatically with no need for other outside controls.

The data demodulation and modulation structures were all designed with a digital receiver structure based on software-defined radio technology. In this model, when demodulated, the radio-frequency (RF) signals are firstly mixed by the carrier generated by PDB and then converted to digital signals by the device analog-digital converter (ADC). Next, they are mixed and transformed into baseband signals by the procedure of digital down-conversion. Finally, the signals at the optimal sampling points are decided and all of the demodulation data bits are stored in data storage devices. The modulation procedure can be seen as having a reverse order to the demodulation procedure, and it will transform data bits into RF signals.

The ideal performance of a communication system can be measured by the CP. The CP of a bandwidth of 1Hz can be calculated as [5]

(1) $$C=\log_{2}\left(1+SNR\right),$$

in which SNR denotes the signal-to-noise ratio (SNR).

(2) $$SNR=\frac{P_{receive}}{P_{noise}},$$

where $P_{receive}$ represents the receiving power at the antenna and $P_{noise}$ is the power of noise at the PDB.

The energy harvesting and delivery structures are designed based on the energy conversion circuits. An impedance matching procedure is used to guarantee that maximum energy transfer efficiency can be reached. When harvesting energy, because the receiving RF signals are alternating current (AC), a module called voltage multiplier is used to transform AC signals into direct current (DC). In addition, to keep the energy storage devices being charged smoothly, a capacitor is used. When delivering energy, a procedure of transmitting sine signals carrying no data occurs. A basic sine signal is generated from the frequency oscillator, and its power is increased by the power amplifier module.

There must be power loss during wireless charging signal propagation, and the energy remaining ratio is closely related to the distance. A model that is widely used for the energy remaining ratio is

(3) $$\xi_{ch}\left(d\right)=\beta_{ch}\cdot {\left(d\right)}^{-\alpha_{ch}},$$

where $\beta_{ch}$ represents the energy remaining ratio at a standard distance of 1 m, and $\alpha_{ch}$ influences the correlation with the distance [19].

### 2.2. LIS Technology

**Figure 3:**
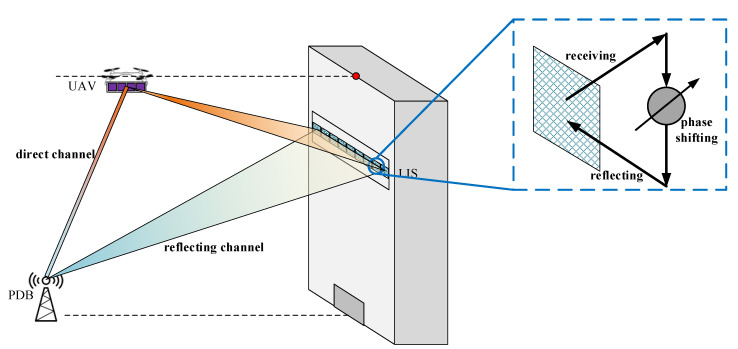
The scene of LIS-assisted multiple input single output (MISO) communication.

LIS technology is an effective and practical type of technology that is used in future wireless communication systems [22]. A widely researched mode used in communication systems is the multiple input single output (MISO) [13,15,16]. The configuration of an LIS in a MISO mode is shown in Figure 3.

The UAV is equipped with an antenna array, so it is possible to for it to communicate with the LIS and PDB simultaneously. The LIS is also an antenna array, which receives the signals from the UAV and reflects them to the PDB. The structure of the reflecting units in the LIS is specially designed, and it is an energy-saving structure. Each array unit can be seen as a circuit containing three components: the receiving part, phase-shifting part, and reflecting part [23].

We assume that the numbers of data transmitting antennas of the UAV, LIS, and PDB are *M*, *N*, and 1. Thus, the channel matrix from the UAV to the PDB can be denoted as g with dimensions of M×1. The channel matrix from the UAV to the LIS can be denoted as H with dimensions of M×N. The channel matrix from the LIS to the PDB can be denoted as h with dimensions of N×1. Because of the structure of the LIS, the phase-shifting procedure can be seen as a diagonal matrix Φ with dimensions of N×N. If we ignore the path loss, H and h have standard complex Gaussian form in Rayleigh channels and are composed of normalized complex elements in Gaussian channels [15]. The phase-shifting matrix is composed of normalized complex elements.

In addition, when considering signal propagation, path loss must exist, and we can simply denote the energy-remaining ratios $\eta_{H}$, $\eta_{h}$, and $\eta_{g}$. If the transmission power of UAV is *P*, and transmission data are *s*, we can model the scene in Figure 3 as [13]

(4) $$\begin{array}{c} r=\sqrt{P}\left(\sqrt{\eta_{H}}\sqrt{\eta_{h}}h\Phi H+\sqrt{\eta_{g}}g\right)f^{H}s+w\\ \;\overset{\Delta }{\;=}\sqrt{P}Qf^{H}s+w, \end{array}$$

where *w* is the channel noise and f is the precoding vector with

(5) $$f=\frac{Q}{∥Q∥}.$$

According to the information theory, the CP of the communication system is closely related to $E\left\{{∥Q∥}^{2}\right\}$. Because of the independence of the channel matrices, we have

(6) $$E\left\{{∥Q∥}^{2}\right\}=\eta_{g}E\left\{{∥g∥}^{2}\right\}+\eta_{H}\eta_{h}E\left\{{∥h\Phi H∥}^{2}\right\}.$$

Next, we consider the two terms in Equation (6), respectively, under conditions of Gaussian channels or Rayleigh channels. We can easily see that in both channel conditions, the first term is

(7) $$E\left\{{∥g∥}^{2}\right\}=M.$$

If the channel between the UAV and LIS is Gaussian and that between the LIS and PDB is Rayleigh, we have

(8) $$E\left\{{∥h\Phi H∥}^{2}\right\}=E\left\{h\Phi HH^{H}\Phi^{H}h^{H}\right\}={∥H∥}_{F}^{2}=MN.$$

Besides, if both channels are Gaussian, the chosen value of Φ is vital. It is obvious that

(9) $$E\left\{{∥h\Phi H∥}^{2}\right\}\leq MN^{2}.$$

It has been proven that, if the phase-shifting values are selected reasonably, then the equal sign of the in Equation (9) can be taken [15]. In other words,

(10) $$E\left\{{∥h\Phi H∥}^{2}\right\}=MN^{2}.$$

Referring to the formula of the CP, shown in Equcation (1), it can be concluded that by introducing an LIS, the SNR will increase and the CP will also increase accordingly. This is because the LIS channel introduces a new positive term into the calculation of $E\left\{{∥Q∥}^{2}\right\}$. Thus, the time cost of the data transmitting procedure will decrease, which is beneficial to the whole task.

### 2.3. Wireless Channel Models in Data Transmitting

In this paper, we discuss a typical urban scene that includes air-to-ground (ATG) propagation from a UAV to all PDBs. Because of heavy traffic and crowds, the straight-line paths between transmitters and receivers are sometimes blocked. If there is no blockage on the straight line between a transmitter and a receiver, then the communication scene is line-of-sight (LOS). On the contrary, if the straight-line path is obstructed and the receiver only receives scattered signals, the scene is non-line-of-sight (NLOS).

**Figure 4:**
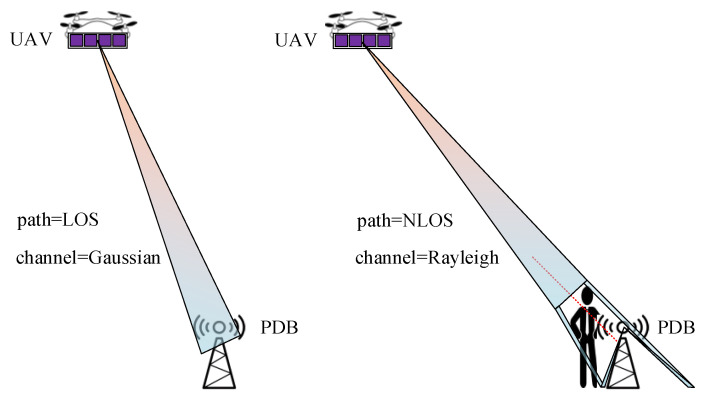
Line-of-sight (LOS) and non-line-of-sight (NLOS) scenes in a communication system.

The two different scenes are shown in Figure 4.

Now, we discuss the path loss model in relation to data transmission. We mentioned two kinds of channels in the previous subsection: Gaussian channels and Rayleigh channels. Actually, in communication scenes, they are related to whether the conditions are LOS or NLOS. If the path is LOS, the channel can be approximately regarded as Gaussian, and if the path is NLOS, the channel is Rayleigh [24]. It is obvious that if the UAV is high in the air directly over the PDB, the probability of blockage occurring is the lowest. A model describing the probability of LOS can be approximated by a sigmoid function [11], which is given by

(11) $$p^{LOS}\left(\theta \right)=\frac{1}{1+a\exp\left[-b\left(\theta -a\right)\right]},$$

where *a* and *b* are parameters that need to be estimated by experiments, and θ is the acute angle between the straight line from the UAV to the PDB and the ground. Then, the probability of NLOS is

(12) $$p^{NLOS}\left(\theta \right)=1-p^{LOS}\left(\theta \right)=\frac{a\exp\left[-b\left(\theta -a\right)\right]}{1+a\exp\left[-b\left(\theta -a\right)\right]}.$$

When an electromagnetic wave propagates in space, its power decreases. An exponential decay model is most commonly used. Similar to the energy delivering procedure given in Equcation (3), the model of the power remaining ratio under LOS conditions can be

(13) $$\xi_{d}^{LOS}\left(d\right)=\beta_{d}\cdot {\left(d\right)}^{-\alpha_{d}},$$

where $\beta_{d}$ represents the power remaining ratio at a standard distance of 1 m, and $\alpha_{d}$ influences the correlation with the distance. In addition, if the signal path is NLOS, the power will certainly decrease and the power remaining ratio can be modeled as multiplying a positive constant of ρ less than 1 [8]. We have

(14) $$\xi_{d}^{NLOS}\left(d\right)=\rho \cdot \beta_{d}\cdot {\left(d\right)}^{-\alpha_{d}},$$

where 0<ρ<1 and can also be estimated by experiments [8].

## 3. System Model and Problem Formulation

### 3.1. System Model

As illustrated in Figure 1, to simplify the problem, we assume that the trajectory of the UAV is along the *x*-axis [7]. There are *K* PDBs deployed between the two buildings, and around each PDB, *I* RNs exist. All RNs around each PDB are uniformly distributed in a circular region with a radius of $a_{0}$. In the task of downlink data transmission, the amount of data that each RN needs to gain is $D_{0}$ bits/Hz.

The whole system operates via TDMA. All RNs around a PDB will be turned on and off at the same time, and the operating order of all PDBs is serial. An omnidirectional antenna is used on each PDB because it is impossible for a PDB to locate all RNs and the UAV before the PDB is charged. When the RNs are being charged, they return energy backwards to the PDB with a ratio of κ, and after the charging procedure of RNs has occurred, each PDB also transmits the data to all RNs serially. The power values of the circuit boards of the RN, PDB, and UAV are $p_{R}$, $p_{B}$, and $p_{U}$. The UAV flight power and the velocity are $p_{fly}$, and *v*.

**Figure 5:**
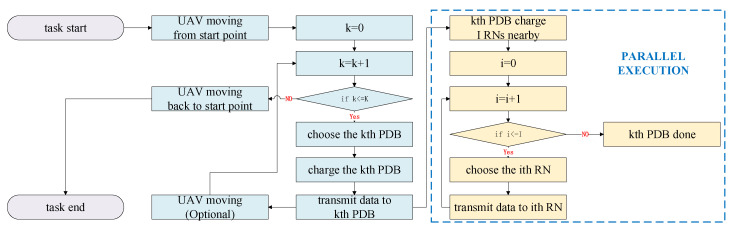
The flow chart of the downlink data transmitting task.

When the data are transmitted from the UAV to each PDB, three channel paths exist: a direct channel and two LIS reflecting channels. There are *M* antennas on the UAV for data transmission, and on each LIS, there are *N* antennas for signal reflection. In addition, a directional antenna with a gain of $G_{0}$ is used on the UAV for energy delivery. A flow chart of the downlink data transmitting task is shown in Figure 5.

The whole downlink data transmission task can be summed up by four procedures: energy and data transmission from the PDB to the RN, data transmission from the UAV to the PDB, energy transmission from the UAV to the PDB, and the UAV flying procedure. Then, if the UAV hovering position is determined, the energy cost and time cost of the whole task are subsequently determined. Referring to the basic technologies section, the whole task can be modeled as a mathematical model based on analytical expressions. Firstly, we consider the procedure of energy and data transmission from the PDB to the RN and derive the expression of the full energy requirement of the *k*-th PDB. Secondly, we consider the procedure of data transmission from the UAV to the PDB and obtain the mean CP, which affects the data transmission rate and data transmission time. Thirdly, we consider the last two procedures and derive the expressions for the total energy cost and total time cost, which are used to construct optimization problems to achieve optimal performance.

### 3.2. Energy and Data Transmission from the PDB to the RN

Without loss of generality, we focus on the *k*-th PDB and discuss the procedure of energy and data transmission from the PDB to all *I* RNs nearby. The receiving power of the communication signal of the *i*th RN is

(15) $$P_{d,BR,r}^{k,i}=P_{d,BR,t}^{k}\cdot \xi_{d}\left(d_{BR}^{i}\right),$$

where $P_{d,BR,t}^{k}$ is the transmission power of the *k*-th PDB, and

(16) $$\xi_{d}\left(d_{BR}^{i}\right)=\beta_{d}\cdot {\left(d_{BR}^{i}\right)}^{-\alpha_{d}}$$

is the path loss function when the communication signal is transmitted.

Then, the SNRs of all RNs nearby is

(17) $$SNR^{k}\left(d_{BR}^{i}\right)=\frac{P_{d,BR,t}^{k}\cdot \beta_{d}\cdot {\left(d_{BR}^{i}\right)}^{-\alpha_{d}}}{\sigma_{R}^{2}},$$

where $\sigma_{R}^{2}$ is the power of additive white Gaussian noise (AWGN) at the RN.

The total communication time from each PDB to all RNs nearby is [25]

(18) $$T_{d,B}^{k}=\sum_{i=1}^{N}T_{d,R}^{k,i}=\sum_{i=1}^{I}\frac{D_{0}}{C_{BR}^{k,i}}=\sum_{i=1}^{I}\frac{D_{0}}{\log_{2}\left(1+SNR^{k}\left(d_{BR}^{i}\right)\right)}.$$

In our model, the value of *I* is large, so we approximate Equcation (18), and we have

(19) $$T_{d,B}^{k}=I\cdot E\left[\frac{D_{0}}{\log_{2}\left(1+SNR^{k}\left(d_{BR}^{i}\right)\right)}\right].$$

The total operation time of the *k*-th PDB is the summation of the energy delivery time and data transmitting time, which is given by

(20) $$T_{all,B}^{k}=T_{ch,B}^{k}+T_{d,B}^{k}.$$

Now, we consider the wireless charging procedure. The receiving power of the charging signal $P_{ch,BR,r}^{k,i}$ is

(21) $$P_{ch,BR,r}^{k,i}=P_{ch,BR,t}^{k}\cdot \xi_{ch}\left(d_{BR}^{i}\right),$$

where $P_{ch,BR,t}^{k}$ is the transmission power of the charging signal of the *k*-th PDB, and

(22) $$\xi_{ch}\left(d_{BR}^{i}\right)=\beta_{ch}\cdot {\left(d_{BR}^{i}\right)}^{-\alpha_{ch}}.$$

Because all *I* RNs run at the same time, the operation time of each RN equals the total operation time $T_{all,B}^{k}$. The energy supply inequation can be derived as

(23) $$p_{R}\cdot T_{all,B}^{k}\leq P_{ch,BR,r}^{k,i}\cdot \left(1-\kappa \right)\cdot T_{ch,B}^{k}.$$

To minimize the time and energy consumption, inequality (23) should be taken at the equal sign when an RN is located on the boundary of the distribution region—i.e., we choose the distance $a_{0}$. Then, we have

(24) $$p_{R}\cdot T_{all,B}^{k}=P_{ch,BR,r}^{k,distance=a_{0}}\cdot \left(1-\kappa \right)\cdot T_{ch,B}^{k},$$

(25) $$P_{ch,BR,r}^{k,distance=a_{0}}=P_{ch,BR,t}^{k}\cdot \xi_{ch}\left(a_{0}\right),$$

and

(26) $$\xi_{ch}\left(a_{0}\right)=\beta_{ch}\cdot {\left(a_{0}\right)}^{-\alpha_{ch}}.$$

Referring to Equations (20) and (24), we can obtain $T_{all,B}^{k}$ and $T_{ch,B}^{k}$ as

(27) $$T_{all,B}^{k}=\frac{P_{ch,BR,r}^{k,distance=a_{0}}\cdot \left(1-\kappa \right)\cdot T_{d,B}^{k}}{P_{ch,BR,r}^{k,distance=a_{0}}\cdot \left(1-\kappa \right)-p_{R}},$$

(28) $$T_{ch,B}^{k}=\frac{p_{R}\cdot T_{d,B}^{k}}{P_{ch,BR,r}^{k,distance}\cdot \left(1-\kappa \right)-p_{R}}.$$

The total energy cost of the *k*-th PDB can be calculated by

(29) $$\begin{array}{c} E_{all,B}^{k}=E_{ch,B}^{k}+E_{d,B}^{k}+E_{c,B}^{k}\\ \;\;\;\;\;=P_{ch,BR,t}^{k}\cdot T_{ch,B}^{k}+P_{d,BR,t}^{k}\cdot T_{d,B}^{k}+p_{B}\cdot T_{all,B}^{k}. \end{array}$$

Finally, we have

(30) $$\begin{array}{c} E_{all,B}^{k}=P_{ch,BR,t}^{k}\cdot T_{ch,B}^{k}+P_{d,BR,t}^{k}\cdot T_{d,B}^{k}+p_{B}\cdot \left(T_{ch,B}^{k}+T_{d,B}^{k}\right)\\ =\left(P_{ch,BR,t}^{k}+p_{B}\right)\cdot T_{ch,B}^{k}+\left(P_{d,BR,t}^{k}+p_{B}\right)\cdot T_{d,B}^{k}\\ =\left[\left(P_{ch,BR,t}^{k}+p_{B}\right)\cdot \frac{p_{R}}{P_{ch,BR,r}^{k,distance}\cdot \left(1-\kappa \right)-p_{R}}+P_{d,BR,t}^{k}+p_{B}\right]\cdot T_{d,B}^{k}\\ =\left[\left(P_{ch,BR,t}^{k}+p_{B}\right)\cdot \frac{p_{R}}{P_{ch,BR,t}^{k}\cdot \xi_{ch}\left(a_{0}\right)\cdot \left(1-\kappa \right)-p_{R}}+P_{d,BR,t}^{k}+p_{B}\right]\cdot I\cdot E\left[\frac{D_{0}}{\log_{2}\left(1+SNR^{k}\left(d_{BR}^{i}\right)\right)}\right]\\ =\left[\left(P_{ch,BR,t}^{k}+p_{B}\right)\cdot \frac{p_{R}}{P_{ch,BR,t}^{k}\cdot \beta_{ch}\cdot {\left(a_{0}\right)}^{-\alpha_{ch}}-p_{R}}+P_{d,BR,t}^{k}+p_{B}\right]\cdot I\cdot E\left[\frac{D_{0}}{\log_{2}\left(1+\frac{P_{d,BR,t}^{k}\cdot \beta_{d}\cdot {\left(d_{BR}^{i}\right)}^{-\alpha_{d}}}{\sigma_{R}^{2}}\right)}\right]. \end{array}$$

### 3.3. Data Transmission from the UAV to the PDB

Now, we consider the data transmitting procedure from the UAV to the *k*-th PDB and calculate the CP of the whole channel. There are three sub-channels from the UAV to a PDB and, as shown in Figure 1, we call them the left channel, right channel, and direct channel. We have stated that all the channels are probably LOS. Hence, we denote the LOS probability of the above channels as $p_{left}^{LOS}$, $p_{right}^{LOS}$ and $p_{direct}^{LOS}$. Similarly, the probabilities of channels being NLOS are $p_{left}^{NLOS}$, $p_{right}^{NLOS}$ and $p_{direct}^{NLOS}$. It is obvious that [8]

(31) $$p_{left}^{LOS}+p_{left}^{NLOS}=1,$$

(32) $$p_{right}^{LOS}+p_{right}^{NLOS}=1,$$

(33) $$p_{direct}^{LOS}+p_{direct}^{NLOS}=1.$$

The overall channel conditions have eight different combinations, and all of them are listed in Table 1.

We can list the receiving signals of all the eight different channel conditions. The transmission data are expressed by *s*, the AWGN at the PDB is expressed by *w*, the receiving data are expressed by *r*, the data transmission power of the UAV is expressed by $P_{d,UB,t}$, and the pre-coding vector of the transmission signal is expressed by f. If a sub-channel is LOS, its path loss is denoted as $\eta_{\bar{H}}$, $\eta_{\bar{h}}$ or $\eta_{\bar{g}}$. If a sub-channel is NLOS, its path loss is denoted as $\eta_{\tilde{h}}$ or $\eta_{\tilde{g}}$, respectively. In addition, we use the angle markers *L* and *R* to represent left and right LIS sub-channels. Thus, we have

(34) $$r=\left\{\begin{array}{c} \sqrt{P_{d,UB,t}}\left[\sqrt{\eta_{{\bar{H}}_{L}}}\sqrt{\eta_{{\bar{h}}_{L}}}{\bar{h}}_{L}\Phi_{L}{\bar{H}}_{L}+\sqrt{\eta_{{\bar{H}}_{R}}}\sqrt{\eta_{{\bar{h}}_{R}}}{\bar{h}}_{R}\Phi_{R}{\bar{H}}_{R}+\sqrt{\eta_{\bar{g}}}\bar{g}\right]f^{H}s+w\;ifL\mathrm{OS},\mathrm{LOS},\mathrm{LOS}\\ \sqrt{P_{d,UB,t}}\left[\sqrt{\eta_{{\bar{H}}_{L}}}\sqrt{\eta_{{\tilde{h}}_{L}}}{\tilde{h}}_{L}\Phi_{L}{\bar{H}}_{L}+\sqrt{\eta_{{\bar{H}}_{R}}}\sqrt{\eta_{{\bar{h}}_{R}}}{\bar{h}}_{R}\Phi_{R}{\bar{H}}_{R}+\sqrt{\eta_{\bar{g}}}\bar{g}\right]f^{H}s+w\;if\mathrm{NLOS},\mathrm{LOS},\mathrm{LOS}\\ \sqrt{P_{d,UB,t}}\left[\sqrt{\eta_{{\bar{H}}_{L}}}\sqrt{\eta_{{\bar{h}}_{L}}}{\bar{h}}_{L}\Phi_{L}{\bar{H}}_{L}+\sqrt{\eta_{{\bar{H}}_{R}}}\sqrt{\eta_{{\tilde{h}}_{R}}}{\tilde{h}}_{R}\Phi_{R}{\bar{H}}_{R}+\sqrt{\eta_{\bar{g}}}\bar{g}\right]f^{H}s+w\;if\mathrm{LOS},\mathrm{NLOS},\mathrm{LOS}\\ \sqrt{P_{d,UB,t}}\left[\sqrt{\eta_{{\bar{H}}_{L}}}\sqrt{\eta_{{\tilde{h}}_{L}}}{\tilde{h}}_{L}\Phi_{L}{\bar{H}}_{L}+\sqrt{\eta_{{\bar{H}}_{R}}}\sqrt{\eta_{{\tilde{h}}_{R}}}{\tilde{h}}_{R}\Phi_{R}{\bar{H}}_{R}+\sqrt{\eta_{\bar{g}}}\bar{g}\right]f^{H}s+w\;if\mathrm{NLOS},\mathrm{NLOS},\mathrm{LOS}\\ \sqrt{P_{d,UB,t}}\left[\sqrt{\eta_{{\bar{H}}_{L}}}\sqrt{\eta_{{\bar{h}}_{L}}}{\bar{h}}_{L}\Phi_{L}{\bar{H}}_{L}+\sqrt{\eta_{{\bar{H}}_{R}}}\sqrt{\eta_{{\bar{h}}_{R}}}{\bar{h}}_{R}\Phi_{R}{\bar{H}}_{R}+\sqrt{\eta_{\tilde{g}}}\tilde{g}\right]f^{H}s+w\;ifL\mathrm{OS},\mathrm{LOS},\mathrm{NLOS}\\ \sqrt{P_{d,UB,t}}\left[\sqrt{\eta_{{\bar{H}}_{L}}}\sqrt{\eta_{{\tilde{h}}_{L}}}{\tilde{h}}_{L}\Phi_{L}{\bar{H}}_{L}+\sqrt{\eta_{{\bar{H}}_{R}}}\sqrt{\eta_{{\bar{h}}_{R}}}{\bar{h}}_{R}\Phi_{R}{\bar{H}}_{R}+\sqrt{\eta_{\tilde{g}}}\tilde{g}\right]f^{H}s+w\;if\mathrm{NLOS},\mathrm{LOS},\mathrm{NLOS}\\ \sqrt{P_{d,UB,t}}\left[\sqrt{\eta_{{\bar{H}}_{L}}}\sqrt{\eta_{{\bar{h}}_{L}}}{\bar{h}}_{L}\Phi_{L}{\bar{H}}_{L}+\sqrt{\eta_{{\bar{H}}_{R}}}\sqrt{\eta_{{\tilde{h}}_{R}}}{\tilde{h}}_{R}\Phi_{R}{\bar{H}}_{R}+\sqrt{\eta_{\tilde{g}}}\tilde{g}\right]f^{H}s+w\;if\mathrm{LOS},\mathrm{NLOS},\mathrm{NLOS}\\ \sqrt{P_{d,UB,t}}\left[\sqrt{\eta_{{\bar{H}}_{L}}}\sqrt{\eta_{{\tilde{h}}_{L}}}{\tilde{h}}_{L}\Phi_{L}{\bar{H}}_{L}+\sqrt{\eta_{{\bar{H}}_{R}}}\sqrt{\eta_{{\tilde{h}}_{R}}}{\tilde{h}}_{R}\Phi_{R}{\bar{H}}_{R}+\sqrt{\eta_{\tilde{g}}}\tilde{g}\right]f^{H}s+w\;if\mathrm{NLOS},\mathrm{NLOS},\mathrm{NLOS} \end{array}\right.$$

Equation (34) can be recorded as below for short

(35) $$r=\sqrt{P_{d,UB,t}}Q_{•,•,•}f_{•,•,•}^{H}s+w,$$

where $Q_{•,•,•}$ is the whole channel vector, and the pre-coding vector is

(36) $$f_{•,•,•}\overset{\Delta }{\;=}\frac{Q_{•,•,•}}{∥Q_{•,•,•}∥}.$$

Next, we calculate the mean CP of the whole channel. The CP of each kind of channel conditions is denoted as [25]

(37) $$C_{•,•,•}=E\left\{\log_{2}\left(1+\frac{P_{d,UB,t}}{\sigma_{w}^{2}}{∥Q_{•,•,•}∥}^{2}\right)\right\}\approx \log_{2}\left(1+\frac{P_{d,UB,t}}{\sigma_{w}^{2}}E\left[{∥Q_{•,•,•}∥}^{2}\right]\right).$$

Thus, when all phase-shifting values $\Phi_{L}$ and $\Phi_{R}$ of LISs are well designed, the CPs under eight different channel conditions can be expressed as

(38) $$E\left[{∥Q_{c_{L},c_{R},c_{D}}∥}^{2}\right]=\eta_{{\bar{H}}_{L}}\eta_{c_{L}}MN\cdot \Gamma \left(c_{L}\right)+\eta_{{\bar{H}}_{R}}\eta_{c_{R}}MN\cdot \Gamma \left(c_{R}\right)+\eta_{c_{D}}M,$$

where $c_{L}$, $c_{R}$, and $c_{D}$ represent the channel conditions of the left channel, right channel, and direct channel, respectively, and $\Gamma \left(•\right)$ is denoted as

(39) $$\Gamma \left(•\right)=\left\{\begin{array}{c} N\;\;\;\mathrm{if}\;\mathrm{the}\;\mathrm{channel}\;\mathrm{is}\;\mathrm{LOS}\\ 1\;\mathrm{if}\;\mathrm{the}\;\mathrm{channel}\;\mathrm{is}\;\mathrm{NLOS}. \end{array}\right.$$

The details of the formulas are provided in Appendix A.

Consequently, the mean CP of the whole channel is

(40) $$\begin{array}{c} C_{UB}^{k}=p_{S,S,S}\cdot C_{S,S,S}+p_{N,S,S}\cdot C_{N,S,S}+p_{S,N,S}\cdot C_{S,N,S}+p_{N,N,S}\cdot C_{N,N,S}\\ \;\;\;+p_{S,S,N}\cdot C_{S,S,N}+p_{N,S,N}\cdot C_{N,S,N}+p_{S,N,N}\cdot C_{S,N,N}+p_{N,N,N}\cdot C_{N,N,N}, \end{array}$$

where all parameters are taken from Table 1, Equcations (37) and (38).

### 3.4. Total Energy Cost and Total Time Cost of the Whole Task

Similarly to the previous subsection, we also analyze the energy delivery procedure between the UAV and the *k*-th PDB. The receiving power of the *k*-th PDB can be formulated as [26]

(41) $$P_{ch,UB,r}^{k}=G_{0}\cdot P_{ch,UB,t}\cdot \xi_{ch}\left(d_{UB}^{k}\right),$$

where the path loss is

(42) $$\xi_{ch}\left(d_{UB}^{k}\right)=\beta_{ch}\cdot {\left(d_{UB}^{k}\right)}^{-\alpha_{ch}}.$$

Hence, the time taken for the UAV to charge the *k*-th PDB is

(43) $$T_{ch,U}^{k}=\frac{E_{all,B}^{k}}{P_{ch,UB,r}^{k}}.$$

We can calculate the total energy cost required for the UAV to charge the *k*-th PDB as

(44) $$E_{ch,U}^{k}=P_{ch,UB,t}\cdot T_{ch,U}^{k}.$$

We have already obtained the CP of the whole channel using Equcation (40). Because the precondition of reaching the maximal CP is that the precoding vector in Equcation (36) can be estimated successfully, many pilots need to be transmitted besides valid data [27,28]. We assume that the ratio of valid data to total-transmission data is λ. Then, the time taken for the UAV to transmit a sufficient amount of data to the *k*-th PDB can be derived as

(45) $$T_{d,U}^{k}=\frac{I\cdot D_{0}}{\lambda \cdot C_{UB}^{k}}.$$

The energy cost of transmitting data to the *k*-th PDB is

(46) $$E_{d,U}^{k}=M\cdot P_{d,UB,t}\cdot T_{d,U}^{k}.$$

We can calculate the total energy cost and time cost for the UAV to finish the task of the *k*-th PDB by

(47) $$E_{all,U}^{k}=E_{d,U}^{k}+E_{ch,U}^{k}+p_{U}\cdot T_{all,U}^{k},$$

(48) $$T_{all,U}^{k}=T_{ch,U}^{k}+T_{d,U}^{k}.$$

The total energy cost of the procedure of UAV hovering and processing the data and the energy transmitting task is

(49) $$E_{all,U}=\sum_{k=1}^{K}\left(E_{all,U}^{k}\right).$$

In addition, the total time cost of the previous procedure is

(50) $$T_{all,U}=\sum_{k=1}^{K}T_{all,U}^{k}.$$

Moreover, we take the energy cost of the UAV flying procedure into consideration. If we assume that the distance flown by the UAV throughout the whole task is *L*, then the energy cost of UAV flying is

(51) $$E_{fly}=T_{fly}\cdot p_{fly},$$

where the UAV flying time

(52) $$T_{fly}=\frac{L}{v}.$$

Consequently, the total energy cost and the total time cost of the whole task can be obtained by

(53) $$E=E_{fly}+E_{all,U},$$

and

(54) $$T=T_{fly}+T_{all,U}.$$

Finally, we have

(55) $$\begin{array}{c} E=T_{fly}\cdot p_{fly}+\sum_{k=1}^{K}\left(E_{d,U}^{k}+E_{ch,U}^{k}+p_{U}\cdot T_{all,U}^{k}\right)\\ =\frac{L}{v}\cdot p_{fly}+\sum_{k=1}^{K}\left[M\cdot P_{d,UB,t}\cdot T_{d,U}^{k}+P_{ch,UB,t}\cdot T_{ch,U}^{k}+p_{U}\cdot \left(T_{ch,U}^{k}+T_{d,U}^{k}\right)\right]\\ =\frac{L}{v}\cdot p_{fly}+\sum_{k=1}^{K}\left[\left(P_{ch,UB,t}+p_{U}\right)\cdot T_{ch,U}^{k}+\left(M\cdot P_{d,UB,t}+p_{U}\right)\cdot T_{d,U}^{k}\right]\\ =\frac{L}{v}\cdot p_{fly}+\sum_{k=1}^{K}\left[\left(1+\frac{p_{U}}{P_{ch,UB,t}}\right)\cdot \frac{E_{all,B}^{k}}{G_{0}\cdot \xi_{ch}\left(d_{UB}^{k}\right)}+\left(M\cdot P_{d,UB,t}+p_{U}\right)\cdot \frac{I\cdot D_{0}}{\lambda \cdot C_{UB}^{k}}\right], \end{array}$$

and

(56) $$\begin{array}{c} T=\frac{L}{v}+\sum_{k=1}^{K}T_{all,U}^{k}\\ =\frac{L}{v}+\sum_{k=1}^{K}\left(T_{ch,U}^{k}+T_{d,U}^{k}\right)\\ =\frac{L}{v}+\sum_{k=1}^{K}\left(\frac{E_{all,B}^{k}}{G_{0}\cdot P_{ch,UB,t}\cdot \xi_{ch}\left(d_{UB}^{k}\right)}+\frac{I\cdot D_{0}}{\lambda \cdot C_{UB}^{k}}\right). \end{array}$$

### 3.5. Proposed Optimization Problems

So far, we have obtained the analytical expressions of the total energy cost and total time cost for the whole downlink data transmitting task. The UAV flying distance *L*, the distances between UAV and PDBs $d_{UB}^{k}$, and the CPs between UAV and PDBs $C_{UB}^{k}$ are all affected by the hovering positions of the UAV. In other words, if the UAV’s hovering positions are determined, all theoretical result values can be determined accordingly. Intuitively speaking, a smaller *L*, a smaller $d_{UB}^{k}$, or a larger $C_{UB}^{k}$ will lead to a smaller energy/time cost.

Then, the optimization problem related to minimizing the energy cost can be formulated as having the following two forms: One based on minimizing the total energy consumption of the UAV and the other based on minimizing the total time cost. Both of them are listed below:

(57) minEorminT.

The constrained conditions are only regarding the UAV hovering positions. Actually, if there is only one PDB in the communication system, the optimization problem is a single variable optimization problem. If there is more than one PDB, a hovering scheme containing several hovering positions must be determined. All of the above optimization problems can be solved by numerical algorithms.

## 4. Methodology, Simulation and Analyses

### 4.1. Parameters, Common Assumptions, and Trajectory Planning Algorithms

According to what is shown in Figure 1 and practical application requirements, we can describe the following four actual situations:

- Scene **I**: The UAV is powered by a wired charging circuit and will hover at a fixed point without flying. Only a single PDB is deployed between the two buildings with the position $x_{0}$.
- Scene **II**: The UAV flies from the starting point, hovers at a fixed point for transmitting, and flies back. Only a single PDB is deployed between the two buildings with the position $x_{0}$.
- Scene **III**: The UAV flies from the starting point, hovers at a fixed point for transmitting, and flies back. Several PDBs are deployed with equal spacing and fully cover the region between the two buildings. The position set of all PDBs is $x_{set}$.
- Scene **IV**: The UAV flies from the starting point, hovers at several points for transmitting, and finally flies back. Several PDBs are deployed with equal spacing and fully cover the region between the two buildings. The position set of all PDBs is $x_{set}$.

**Figure 6:**
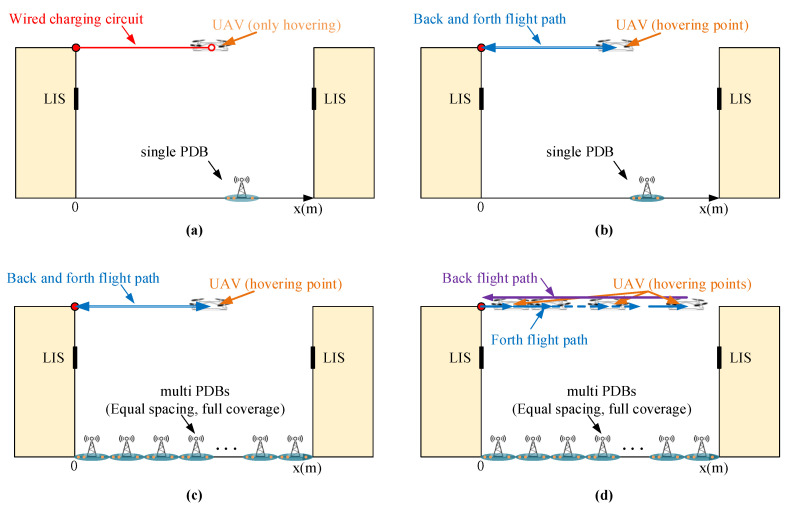
Actual situations of the scene presented in Figure 1: (**a**) Scene **I**, (**b**) Scene **II**, (**c**) Scene **III**, and (**d**) Scene **IV**.

All of the actual situations are shown in Figure 6.

Reasonable parameters were chosen in accordance with the actual environment and several studies [5,7,11,15,21]. We defined the distance between the two buildings as $L_{0}$. We defined the heights of the buildings, LISs, and PDBs as $y_{UAV}$, $y_{LIS}$, and $y_{PDB}$. We defined the transmitting power of all antennas for both data transmission and power delivery at the UAV and all PDBs as $P_{t}$. All fixed parameters, such as $L_{0}$, $y_{UAV}$, $y_{LIS}$, $y_{PDB}$, and $P_{t}$, have to be measured accurately in an actual scene. The supposed parameter values are all listed in Table 2.

Here, we give a common trajectory planning algorithm that can be used to solve the tasks in actual scenes. The optimal solution algorithm is based on a linear search method or a genetic algorithm [29,30]. The two numerical algorithms are listed as Algorithms A1 and A2, and both are listed in Appendix B.

### 4.2. Simulations of the Scenes

The scenes shown in Figure 6 are simulated in the following section. We mainly focused on the energy cost and time cost of the scenes, and all calculations were implemented by MATLAB software.

#### 4.2.1. Scene **I**

**Figure 7:**
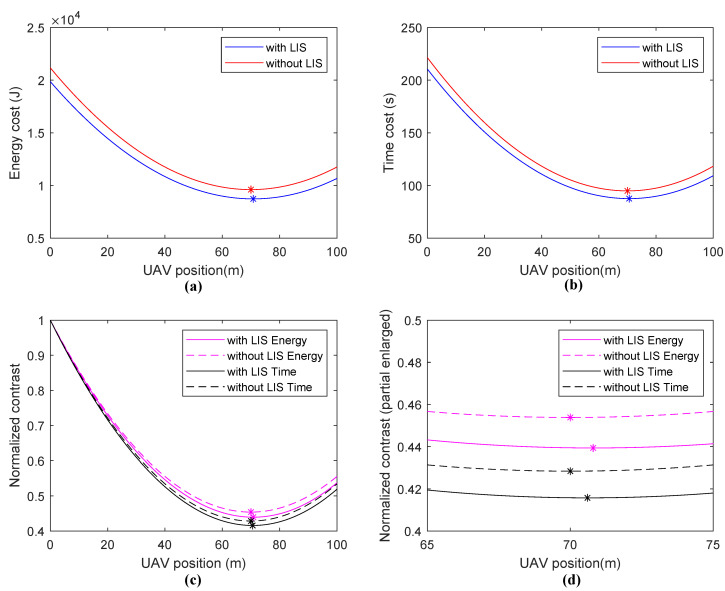
Simulation results of Scene **I**: (**a**) energy cost, (**b**) time cost, (**c**) normalized contrast, and (**d**) normalized contrast (partial enlarged).

In Scene **I**, a fixed PDB is located at a position of 70 m, and the UAV is powered by a wired charging circuit. Thus, it is unnecessary to consider the flying energy cost and time cost, because the UAV can be seen as a stationary aerial base station. We used the linear search method and calculated the total energy cost and time cost of the UAV. The results are shown in Figure 7.

We found that, if the system operates without the assistance of LISs, both the energy cost and the time cost increased. Choosing a reasonable hovering position can achieve a better system performance, because a trade-off exists between the energy/time cost of energy charging and data transmission. An optimal UAV hovering position exists, and if there is no LIS assistance, the best UAV position is the same *x*-axis position as that of the PDB. This result conforms to reality, because when the UAV is right over the PDB, the probability of the LOS reaches a maximum value. In addition, if the system is assisted by LISs, the optimal hovering position is a bit larger than the PDB position under the given parameters. Then, the CP between the UAV and the PDB increases, which accounts for the reduction in data transmitting energy/time cost.

#### 4.2.2. Scene **II**

**Figure 8:**
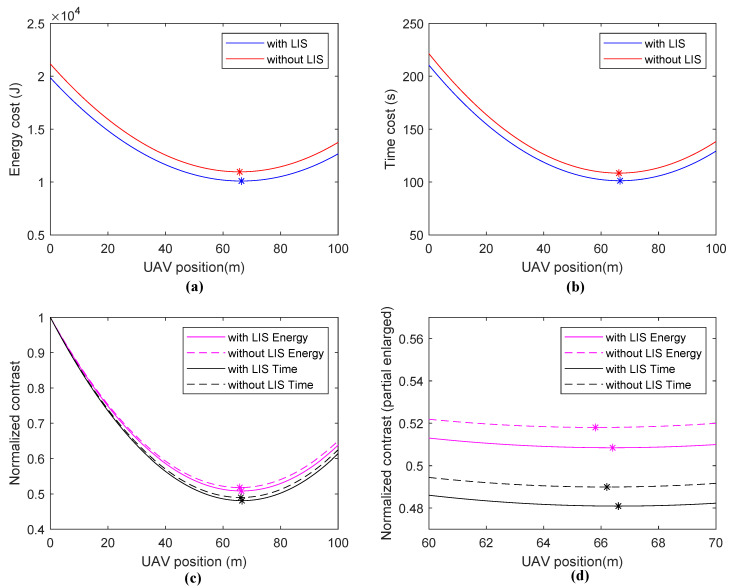
Simulation results of Scene **II**: (**a**) energy cost, (**b**) time cost, (**c**) normalized contrast, and (**d**) normalized contrast (partial enlarged).

In Scene **II**, the flying energy cost and time cost of the UAV are taken into consideration. Accordingly, a trade-off occurs among the energy/time cost of UAV flying, energy charging, and data transmission. Firstly, we also fix the PDB at a position of 70 m. The simulation results are shown in Figure 8. There is a similar result where, with the assistance of LISs, the system performance is improved. However, because of the influence of the UAV flying procedure, all optimal UAV positions are nearer to the starting point than those in Scene **I**. This is mainly for reducing the flight path length of the UAV.

**Figure 9:**
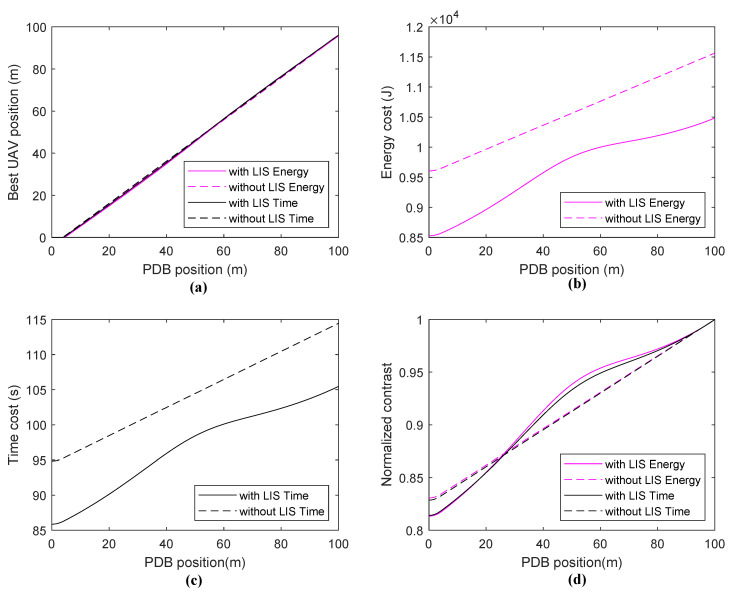
Relationship between the best UAV position and single PDB position in Scene **II**: (**a**) best UAV position, (**b**) energy cost, (**c**) time cost, and (**d**) normalized contrast.

Next, we analyzed the relationship between the best UAV position and the single PDB position. The results are shown in Figure 9. We can draw the conclusion that there is a positive correlation between them based on the parameters given in Table 2. Without the assistance of LISs, an approximately linear increase relationship can be found, because the best UAV position should be the same as that of the PDB, and there is a linear increasing relationship between the UAV flying distance and flying costs. If the system operates with LISs, the relationship becomes nonlinear. This is mainly because of the changes in path loss and LOS probabilities. The energy saving ratio and time saving ratio are all about 11% under the given parameters when the system is assisted by LISs.

#### 4.2.3. Scene **III**

**Figure 10:**
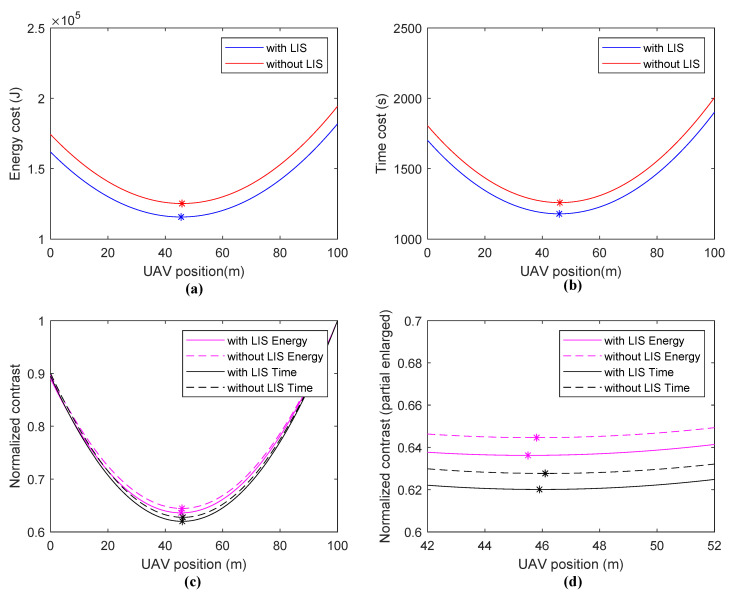
Simulation results of Scene **III**: (**a**) energy cost, (**b**) time cost, (**c**) normalized contrast, and (**d**) normalized contrast (partial enlarged).

In Scene **III**, a full coverage model is analyzed, where all PDBs are located at $\left\{5,15,25,\ldots ,95\right\}$ m. This model is very close to practical application, and the UAV flies from the starting point and hovers at a single point. The energy cost and time cost of the UAV flying procedure are taken into consideration. There are significant performance differences between scenes using LISs and those not using LISs. In addition, the best UAV position is at the left side of the center of two buildings when using the given parameters. This is because if we omit the UAV flying procedure, all components in the system are symmetric about the midline. However, the energy/time cost of UAV flying exists, so the optimal position moves left to balance the UAV flying cost and the energy and data transmitting cost. All solutions are shown in Figure 10.

#### 4.2.4. Scene **IV**

**Figure 11:**
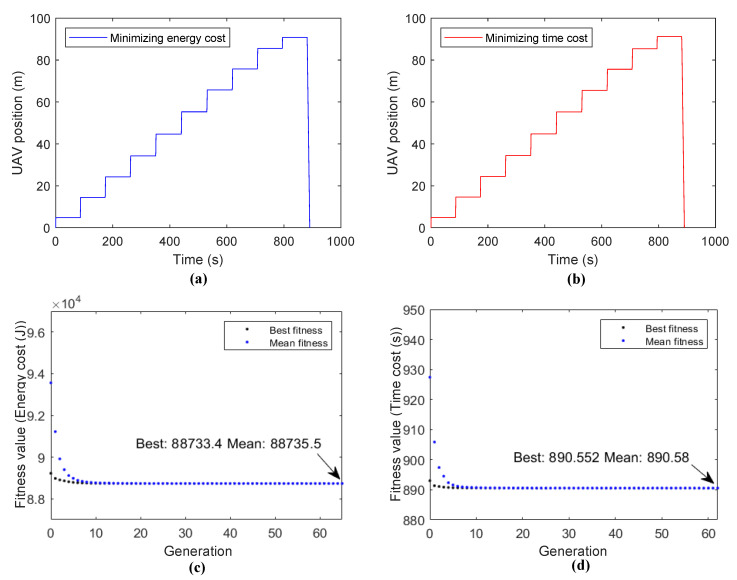
Simulation results of Scene **IV**: (**a**) UAV position at different times when minimizing the energy cost, (**b**) UAV position at different times when minimizing the time cost, (**c**) fitness value of the energy cost at different generations, and (**d**) fitness value of the time cost at different generations.

In Scene **IV**, a more flexible operation mode where the UAV hovers at one point for each PDB is proposed. To maintain the stability of the system, we assume that the UAV flying procedure and energy/data transmitting procedure are separated—i.e., the UAV flies and hovers at a point and then transmits the energy and data to a PDB. As with the result of the previous simulation, for each PDB, only one optimal hovering point exists. It is reasonable to assume that the number of hovering points is the same as the number of PDBs. Then, the UAV flies from the starting point and hovers at all hovering points one by one and flies back after finishing the task with the last PDB at the last hovering point. A genetic-algorithm-based Algorithm A2 is used to determine the best selection of all the hovering points, and all the solutions under LIS-assisted conditions are shown in Figure 11.

We did a simple comparison between the LIS-assisted solutions presented in Scene **III** and Scene **IV** below. When the UAV only hovers at a single point, the minimum energy cost is $1.16\times 10^{5}$ J, and when using the trajectory planning method, the minimum energy becomes $8.87\times 10^{4}$ J. There is an energy saving ratio of about 23.5%. In addition, when the UAV only hovers at a single point, the minimum time cost is 1179.36 s, and when using the trajectory planning method, the minimum energy becomes 890.55 s. There is an energy saving ratio of about 24.5%. Thus, the conclusion can be drawn that reasonable trajectory planning is important to save both energy and time. Such significant performance promotion is beneficial for the scheme where the UAV is arranged to serve all PDBs at different optimal hovering positions. By applying the genetic-algorithm-based Algorithm A2 to solve the optimal hovering positions, a feasible UAV trajectory can be obtained conveniently. Similar conclusions can be extended to systems with PDBs distributed arbitrarily.

### 4.3. Discussion

The simulation results show that if the communication system is assisted by LISs, there is a significant performance improvement in the UAV energy/time cost. In addition, reasonable trajectory planning improves the system’s performance, especially when adopting the strategy of multiple hovering positions. Thus, it is worth noting that, when researching the efficiency promotion of a communication system, considering approaches to enlarge CP, such as using LISs and utilizing ABSs such as UAVs, is important.

An urban scene in which vast IOT nodes are served between buildings was considered in this paper, and the communication channels were all modeled as a widely studied and adopted fitting channel model—i.e., an elevation angle-dependent probabilistic LOS model [8]. Although the model is quasi-accurate, it still does not reflect the measured environment. Future research should take experimental data into consideration after the deployment of 5G communication systems and UAV communication systems.

This paper provides a unified framework that can be used to research the efficiency improvement problems related to UAV communication systems that serve vast IOT nodes. The proposed framework is practical and feasible. By deriving the analytical expressions of the total energy/time required to finish the whole data transmitting task, optimization problems can be obtained with respect to the UAV hovering positions. Thus, by using mathematical tools, the optimal solutions can be determined. In a more complex urban environment, such as one with more UAVs or more buildings, the system models and optimization problems can still be conducted similarly as long as the channel models are measured.

## 5. Conclusions

In this paper, a system of energy and data transmission to vast IOT nodes assisted by UAV, LIS, and PDB is proposed, where a PDB acts as a relay station. The whole system is based on TDMA, and the UAV finishes the task with PDBs one by one. Firstly the UAV delivers energy to a PDB and then transmits data. Similarly, the PDB charges IOT nodes nearby and communicates with them orderly. When analyzing the channels between UAV and a PDB, the existence of LISs deployed on the outside wall of buildings is taken into consideration.

The expressions for the total energy cost and total time cost were derived and optimization problems for minimizing them were proposed. The expressions obtained were related to the hovering points of the UAV and affect the probability of LOS and signal path loss. Four actual scenes that are possible for future communication systems were analyzed. All optimization problems proposed in the scenes were solved by numerical algorithms based on a linear search method or a genetic algorithm. The simulation results show the performance improvement resulting from the deployment of LISs and the importance of planning the UAV trajectory reasonably.

The proposed model and the scenarios are feasible for use in smart cites. Because of the limitations of the experimental conditions, we made some reasonable assumptions for some models and parameters. Further experiments can be performed in the future. In addition, in this paper, all IOT nodes were served by a single UAV, and the UAV flying model was linear. Scenes with multiple UAVs and complex UAV flying models will also be studied in future work. Nevertheless, our research conclusions have strong practical significance, and the research framework can be generalized similarly.

## Figures and Tables

**Table 1:** Eight different combinations of the overall conditions of the channels.

Left Channel	Right Channel	Direct Channel	The Probability Value of the Current Total Channel
LOS	LOS	LOS	$p_{S,S,S}=p_{left}^{LOS}\cdot p_{right}^{LOS}\cdot p_{direct}^{LOS}$
NLOS	LOS	LOS	$p_{N,S,S}=p_{left}^{NLOS}\cdot p_{right}^{LOS}\cdot p_{direct}^{LOS}$
LOS	NLOS	LOS	$p_{S,N,S}=p_{left}^{LOS}\cdot p_{right}^{NLOS}\cdot p_{direct}^{LOS}$
NLOS	NLOS	LOS	$p_{N,N,S}=p_{left}^{NLOS}\cdot p_{right}^{NLOS}\cdot p_{direct}^{LOS}$
LOS	LOS	NLOS	$p_{S,S,N}=p_{left}^{LOS}\cdot p_{right}^{LOS}\cdot p_{direct}^{NLOS}$
NLOS	LOS	NLOS	$p_{N,S,N}=p_{left}^{NLOS}\cdot p_{right}^{LOS}\cdot p_{direct}^{NLOS}$
LOS	NLOS	NLOS	$p_{S,N,N}=p_{left}^{LOS}\cdot p_{right}^{NLOS}\cdot p_{direct}^{NLOS}$
NLOS	NLOS	NLOS	$p_{N,N,N}=p_{left}^{NLOS}\cdot p_{right}^{NLOS}\cdot p_{direct}^{NLOS}$

**Table 2:** Reasonable parameters used in the simulations.

Parameter Symbols	Parameter Values	Parameter Symbols	Parameter Values
$L_{0}$	100 m	$y_{UAV}$	50 m
$y_{LIS}$	40 m	$y_{PDB}$	1 m
$x_{0}$	70 m	$x_{set}$	$\left\{5,15,25,\ldots ,95\right\}$ m
$a_{0}$	5 m	$D_{0}$	1 bit/Hz
*M*	4	*N*	200
*I*	50	*K*	19
$P_{t}$	10 W	$p_{U}$	80 W
$p_{B}$	0.1 W	$p_{R}$	0.001 W
$p_{fly}$	100 W	*v*	10 m/s
$\sigma_{R}^{2}$	$10^{-6}$ W	$\sigma_{w}^{2}$	$10^{-6}$ W
*a*	11.95	*b*	0.136
$\beta_{ch}$	1	$\alpha_{ch}$	2
$\beta_{d}$	1	$\alpha_{d}$	2
ρ	0.5	$G_{0}$	20 dB
λ	0.1	κ	0.1

## Appendix A. Detailed Expressions of $E\left[{∥Q_{c_{L},c_{R},c_{D}}∥}^{2}\right]$

Equation (38) can be written in detail as shown below.

(A1) $$E\left[{∥Q_{S,S,S}∥}^{2}\right]=\eta_{{\bar{H}}_{L}}\eta_{{\bar{h}}_{L}}{∥{\bar{h}}_{L}\Phi_{L}{\bar{H}}_{L}∥}^{2}+\eta_{{\bar{H}}_{R}}\eta_{{\bar{h}}_{R}}{∥{\bar{h}}_{R}\Phi_{R}{\bar{H}}_{R}∥}^{2}+\eta_{\bar{g}}M=\eta_{{\bar{H}}_{L}}\eta_{{\bar{h}}_{L}}MN^{2}+\eta_{{\bar{H}}_{R}}\eta_{{\bar{h}}_{R}}MN^{2}+\eta_{\bar{g}}M$$

(A2) $$E\left[{∥Q_{N,S,S}∥}^{2}\right]=\eta_{{\bar{H}}_{L}}\eta_{{\tilde{h}}_{L}}{∥{\tilde{h}}_{L}\Phi_{L}{\bar{H}}_{L}∥}^{2}+\eta_{{\bar{H}}_{R}}\eta_{{\bar{h}}_{R}}{∥{\bar{h}}_{R}\Phi_{R}{\bar{H}}_{R}∥}^{2}+\eta_{\bar{g}}M=\eta_{{\bar{H}}_{L}}\eta_{{\tilde{h}}_{L}}MN+\eta_{{\bar{H}}_{R}}\eta_{{\bar{h}}_{R}}MN^{2}+\eta_{\bar{g}}M$$

(A3) $$E\left[{∥Q_{S,N,S}∥}^{2}\right]=\eta_{{\bar{H}}_{L}}\eta_{{\bar{h}}_{L}}{∥{\bar{h}}_{L}\Phi_{L}{\bar{H}}_{L}∥}^{2}+\eta_{{\bar{H}}_{R}}\eta_{{\tilde{h}}_{R}}{∥{\tilde{h}}_{R}\Phi_{R}{\bar{H}}_{R}∥}^{2}+\eta_{\bar{g}}M=\eta_{{\bar{H}}_{L}}\eta_{{\bar{h}}_{L}}MN^{2}+\eta_{{\bar{H}}_{R}}\eta_{{\tilde{h}}_{R}}MN+\eta_{\bar{g}}M$$

(A4) $$E\left[{∥Q_{N,N,S}∥}^{2}\right]=\eta_{{\bar{H}}_{L}}\eta_{{\tilde{h}}_{L}}{∥{\tilde{h}}_{L}\Phi_{L}{\bar{H}}_{L}∥}^{2}+\eta_{{\bar{H}}_{R}}\eta_{{\tilde{h}}_{R}}{∥{\tilde{h}}_{R}\Phi_{R}{\bar{H}}_{R}∥}^{2}+\eta_{\bar{g}}M=\eta_{{\bar{H}}_{L}}\eta_{{\tilde{h}}_{L}}MN+\eta_{{\bar{H}}_{R}}\eta_{{\tilde{h}}_{R}}MN+\eta_{\bar{g}}M$$

(A5) $$E\left[{∥Q_{S,S,N}∥}^{2}\right]=\eta_{{\bar{H}}_{L}}\eta_{{\bar{h}}_{L}}{∥{\bar{h}}_{L}\Phi_{L}{\bar{H}}_{L}∥}^{2}+\eta_{{\bar{H}}_{R}}\eta_{{\bar{h}}_{R}}{∥{\bar{h}}_{R}\Phi_{R}{\bar{H}}_{R}∥}^{2}+\eta_{\tilde{g}}M=\eta_{{\bar{H}}_{L}}\eta_{{\bar{h}}_{L}}MN^{2}+\eta_{{\bar{H}}_{R}}\eta_{{\bar{h}}_{R}}MN^{2}+\eta_{\tilde{g}}M$$

(A6) $$E\left[{∥Q_{N,S,N}∥}^{2}\right]=\eta_{{\bar{H}}_{L}}\eta_{{\tilde{h}}_{L}}{∥{\tilde{h}}_{L}\Phi_{L}{\bar{H}}_{L}∥}^{2}+\eta_{{\bar{H}}_{R}}\eta_{{\bar{h}}_{R}}{∥{\bar{h}}_{R}\Phi_{R}{\bar{H}}_{R}∥}^{2}+\eta_{\tilde{g}}M=\eta_{{\bar{H}}_{L}}\eta_{{\tilde{h}}_{L}}MN+\eta_{{\bar{H}}_{R}}\eta_{{\bar{h}}_{R}}MN^{2}+\eta_{\tilde{g}}M$$

(A7) $$E\left[{∥Q_{S,N,N}∥}^{2}\right]=\eta_{{\bar{H}}_{L}}\eta_{{\bar{h}}_{L}}{∥{\bar{h}}_{L}\Phi_{L}{\bar{H}}_{L}∥}^{2}+\eta_{{\bar{H}}_{R}}\eta_{{\tilde{h}}_{R}}{∥{\tilde{h}}_{R}\Phi_{R}{\bar{H}}_{R}∥}^{2}+\eta_{\tilde{g}}M=\eta_{{\bar{H}}_{L}}\eta_{{\bar{h}}_{L}}MN^{2}+\eta_{{\bar{H}}_{R}}\eta_{{\tilde{h}}_{R}}MN+\eta_{\tilde{g}}M$$

(A8) $$E\left[{∥Q_{N,N,N}∥}^{2}\right]=\eta_{{\bar{H}}_{L}}\eta_{{\tilde{h}}_{L}}{∥{\tilde{h}}_{L}\Phi_{L}{\bar{H}}_{L}∥}^{2}+\eta_{{\bar{H}}_{R}}\eta_{{\tilde{h}}_{R}}{∥{\tilde{h}}_{R}\Phi_{R}{\bar{H}}_{R}∥}^{2}+\eta_{\tilde{g}}M=\eta_{{\bar{H}}_{L}}\eta_{{\tilde{h}}_{L}}MN+\eta_{{\bar{H}}_{R}}\eta_{{\tilde{h}}_{R}}MN+\eta_{\tilde{g}}M$$

## Appendix B. Trajectory Planning Algorithms

The two numerical algorithms used for solving the trajectory planning tasks are listed as below. Algorithm A1 is based on the linear search method, and Algorithm A2 is based on the genetic algorithm. When used for simulations, all the algorithms were implemented by MATLAB software.

**Algorithm A1:** Optimal solution algorithm used to solve Equation (57) for Scene **I**-**III**

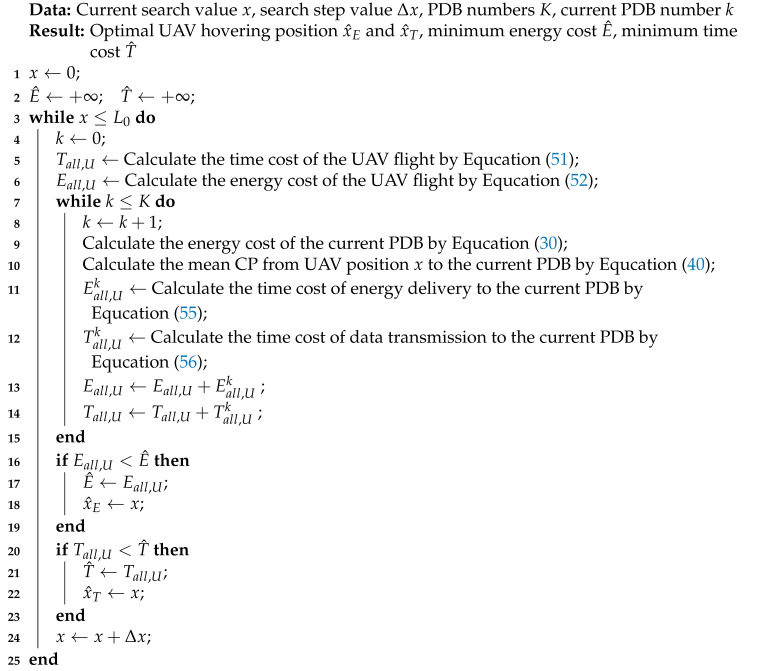

**Algorithm A2:** Optimal solution algorithm to solve Equation (57) for Scene **IV**

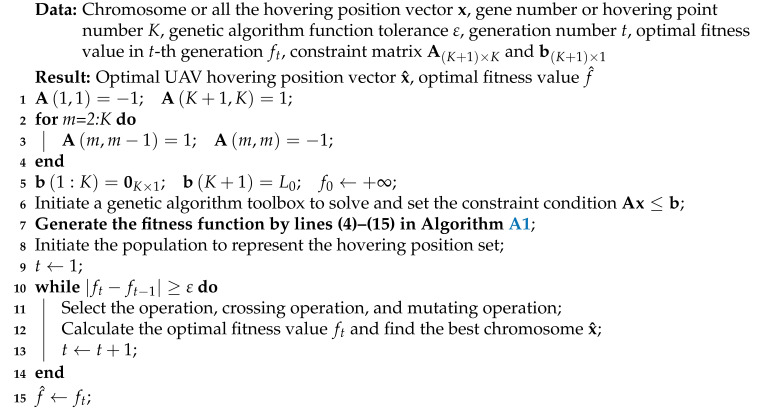

The complexity of the above algorithms is briefly analyzed as follows. Algorithm A1 is used for single hovering points, and we define the ratio of $L_{0}$ and Δx as *n*, which is the number of search points. In addition, the operation used to calculate the total energy cost and total time cost of a PDB is *c*, and it is obvious that *c* is constant, because the calculation procedure is uniform at different UAV hovering positions. Then, the complexity of Algorithm A1 is $o\left(Kcn\right)$, which mainly depends on the searching precision Δx. However, if a similar search method is used to solve the problem of multiple hovering positions with the number of hovering positions being *l*, the complexity can be $o\left(K{cn}^{l}\right)$. The complexity of the algorithm increases exponentially, so we propose a genetic-algorithm-based Algorithm A2 to solve such scenes. The complexity becomes $o\left(Kcvpl\right)$, where *v* is the maximum number of iterations and *p* is the population number. Actually, the computational complexity is reduced by using Algorithm A2 when *l* is a large number.

## References

[1] Abbas G., Abbas Z.H., Waqas M., Hassan A.K. (2020). Spectrum utilization efficiency in the cognitive radio enabled 5G-based IoT. J. Netw. Comput. Appl..

[2] Hellaoui H., Koudil M., Bouabdallah A. (2020). Energy Efficiency in Security of 5G-Based IoT: An End-to-End Adaptive Approach. IEEE Internet Things J..

[3] Cirillo F., Gomez D., Diez L., Elicegui Maestro I., Gilbert T.B.J., Akhavan R. (2020). Smart City IoT Services Creation Through Large-Scale Collaboration. IEEE Internet Things J..

[4] Vedady Moghadam M.R., Zeng Y., Zhang R. Waveform optimization for radio-frequency wireless power transfer: (Invited paper). Proceedings of the 2017 IEEE 18th International Workshop on Signal Processing Advances in Wireless Communications (SPAWC).

[5] Lu X., Wang P., Niyato D., Kim D.I., Han Z. (2017). Wireless Networks with RF Energy Harvesting: A Contemporary Survey. IEEE Commun. Surv. Tutorials.

[6] Kim T., Park H., Jung Y., Lee S. (2020). Wi-Fi Backscatter System with Tag Sensors Using Multi-Antennas for Increased Data Rate and Reliability. Sensors.

[7] Yang S., Deng Y., Tang X., Ding Y., Zhou J. (2019). Energy Efficiency Optimization for UAV-Assisted Backscatter Communications. IEEE Commun. Lett..

[8] Zeng Y., Wu Q., Zhang R. (2019). Accessing From the Sky: A Tutorial on UAV Communications for 5G and Beyond. Proc. IEEE.

[9] Alsaeedy A., Chong E. (2020). 5G and UAVs for Mission-Critical Communications: Swift Network Recovery for Search-and-Rescue Operations. Mob. Networks Appl..

[10] Lu J., Wan S., Chen X., Fan P. Energy-Efficient 3D UAV-BS Placement versus Mobile Users’ Density and Circuit Power. Proceedings of the 2017 IEEE Globecom Workshops (GC Wkshps).

[11] Al-Hourani A., Kandeepan S., Lardner S. (2014). Optimal LAP Altitude for Maximum Coverage. IEEE Wirel. Commun. Lett..

[12] Feng W., Wang J., Chen Y., Wang X., Ge N., Lu J. (2019). UAV-Aided MIMO Communications for 5G Internet of Things. IEEE Internet Things J..

[13] Li Y., Jiang M., Zhang Q., Qin J. (2019). Joint Beamforming Design in Multi-Cluster MISO NOMA Intelligent Reflecting Surface-Aided Downlink Communication Networks. arXiv.

[14] Yan W., Yuan X., Kuai X. (2020). Passive Beamforming and Information Transfer via Large Intelligent Surface. IEEE Wirel. Commun. Lett..

[15] Han Y., Tang W., Jin S., Wen C., Ma X. (2019). Large Intelligent Surface-Assisted Wireless Communication Exploiting Statistical CSI. IEEE Trans. Veh. Technol..

[16] Huang C., Zappone A., Debbah M., Yuen C. Achievable Rate Maximization by Passive Intelligent Mirrors. Proceedings of the 2018 IEEE International Conference on Acoustics, Speech and Signal Processing (ICASSP).

[17] Mohamed Z., Aissa S. Leveraging UAVs with Intelligent Reflecting Surfaces for Energy-Efficient Communications with Cell-Edge Users. Proceedings of the 2020 IEEE International Conference on Communications Workshops (ICC Workshops).

[18] Xu J., Zeng Y., Zhang R. (2017). UAV-enabled multiuser wireless power transfer: Trajectory design and energy optimization. Proceedings of the 23rd Asia-Pacific Conference on Communications (APCC).

[19] Han K., Huang K. (2017). Wirelessly Powered Backscatter Communication Networks: Modeling, Coverage, and Capacity. IEEE Trans. Wirel. Commun..

[20] Feng M., Mao S., Jiang T. (2018). Dynamic Base Station Sleep Control and RF Chain Activation for Energy-Efficient Millimeter-Wave Cellular Systems. IEEE Trans. Veh. Technol..

[21] Yin S., Zhao Y., Li L. (2019). Resource Allocation and Basestation Placement in Cellular Networks With Wireless Powered UAVs. IEEE Trans. Veh. Technol..

[22] Yuan J., Ngo H.Q., Matthaiou M. (2020). Towards Large Intelligent Surface (LIS)-based Communications. IEEE Trans. Commun..

[23] Wu Q., Zhang R. (2019). Intelligent Reflecting Surface Enhanced Wireless Network: Joint Active and Passive Beamforming Design. IEEE Trans. Commun..

[24] Trigui I., Affes S., Liang B. (2017). Unified Stochastic Geometry Modeling and Analysis of Cellular Networks in LOS/NLOS and Shadowed Fading. IEEE Trans. Wirel. Commun..

[25] Zeng Y., Xu J., Zhang R. (2019). Energy Minimization for Wireless Communication With Rotary-Wing UAV. IEEE Trans. Wirel. Commun..

[26] Venugopal K., Valenti M.C., Heath R.W. (2016). Device-to-Device Millimeter Wave Communications: Interference, Coverage, Rate, and Finite Topologies. IEEE Trans. Wirel. Commun..

[27] Alkhateeb A., El Ayach O., Leus G., Heath R.W. (2014). Channel Estimation and Hybrid Precoding for Millimeter Wave Cellular Systems. IEEE J. Sel. Top. Signal Process..

[28] Fan D., Gao F., Ai B., Wang G., Zhong Z., Deng Y., Nallanathan A. (2019). Channel Estimation and Self-Positioning for UAV Swarm. IEEE Trans. Commun..

[29] Chong E.K.P., Zak S.H. (2013). An Introduction to Optimization. IEEE Antennas Propag. Mag..

[30] Roberge V., Tarbouchi M., Labonte G. (2018). Fast Genetic Algorithm Path Planner for Fixed-Wing Military UAV Using GPU. IEEE Trans. Aerosp. Electron. Syst..

